# Positive association of female overactive bladder symptoms and estrogen deprivation: A nationwide population-based cohort study in Taiwan

**DOI:** 10.1097/MD.0000000000004107

**Published:** 2016-07-18

**Authors:** Chen-Li Cheng, Jian-Ri Li, Ching-Heng Lin, William C. de Groat

**Affiliations:** a Division of Urology, Department of Surgery, Taichung Veterans General Hospital; b Institute of Medicine, Chun Sang Medical University; c Department of Medicine and Nursing, Hungkuang University; d Department of Medical Research, Taichung Veterans General Hospital, Taichung, Taiwan; e Department of Pharmacology and Chemical Biology, University of Pittsburgh School of Medicine, Pittsburgh, Pennsylvania.

**Keywords:** breast cancer, estrogen, lower urinary tract symptoms, overactive bladder, urinary incontinence

## Abstract

**Objective:**

Estrogen is considered to be a unique hormone in females that has an impact on voiding function. Animal models and clinical epidemiologic studies showed high correlation between estrogen deficiency and female overactive bladder (OAB) symptoms. We designed a population-based cohort study from a national health database to assess the association of estrogen deprivation therapy and female OAB.

**Materials and methods:**

This study examined the records of 16,128 patients ranging in age from 18 to 40 that were included in the Taiwan National Health Insurance Research Database (NHIRD) in the years between 2001 and 2010. Of these, 1008 had breast cancer with hormone therapy only and the other 15,120 controls did not have breast cancer or hormone therapy. All patients with neurologic diseases and those with pre-existing OAB identified by information in the NHIRD database were excluded. OAB was defined by medications prescribed for at least 1 month. Risk of new onset OAB in the breast cancer and nonbreast cancer groups was estimated. Fourteen patients (1.4%) experienced OAB in the breast cancer group. Overall, breast cancer with estrogen deprivation therapy increased the risk of OAB by 14.37-fold (adjusted hazard ratio, 95% confidence interval 7.06–29.27). Subgroup analysis showed that in the older age breast cancer group (36–40), a lower Charlson comorbidity index (CCI) score and antidepressant medication use for at least 30 days had an impact on the increase of OAB risk. After adjustment of variables, the higher CCI and the use of antipsychotic drugs increased risk of OAB 3.45-fold and 7.45-fold, respectively. The Kaplan–Meier analysis of OAB-free survival in the breast cancer group showed a significant time-dependent increase in incidence of OAB.

**Conclusion:**

Estrogen deprivation in young patients with breast cancer increased the risk of OAB. The OAB development rate was steady and fast in the beginning 3 years after estrogen deprivation. This result indicates a role of estrogen in the modulation of female voiding function.

## 1 Introduction

Symptoms of lower urinary tract dysfunction can occur during urine storage, voiding and postvoiding period.^[1]^ In females, bladder storage symptoms including urinary frequency, urgency with or without urinary incontinence (UI), and nocturia account for the majority of bladder-related complaints.^[2–6]^ These symptoms are largely encompassed by the term overactive bladder (OAB) and have a severe impact on the quality of life.

Human epidemiology studies revealed that the prevalence of female OAB is 20.9% with an age-dependent increase to 34.5% in those who were over the age of 65.^[2]^ The impact of aging on OAB and UI may be related in part to changes in estrogen levels.^[7]^ Estrogen deprivation in the ovariectomy (OVX) rat model decreases voiding efficiency and increases postvoiding volume.^[8,9]^ Because there are no well-designed epidemiologic studies that have examined the association of estrogen and female OAB, we have used a nationwide population-based insurance database to assess this possible association.

In clinical practice, both general physicians and urologists prescribe medications to decrease bladder contractions in order to reduce OAB or UI symptoms. Thus, patients receiving prescriptions for these medications over a prolonged period would be considered to have OAB. In the present study, we used this information to identify patients exhibiting OAB symptoms.

## 2 Materials and methods

### 2.1 Data sources

All data used in this study were retrieved from the Taiwan National Health Insurance Research Database (NHIRD), which is managed by the Taiwan National Health Research Institute (NHRI). The Taiwan National Health Insurance program covers 99% of the population of the island since 1995. In this study, we used a systemic sampling of data from the Taiwan NHIRD from 2001 to 2010 with a total of 1,000,000 individuals. These random subjects have been approved by the NHRI to be representative of the general population in Taiwan. Besides, this study protocols were accordance to the guideline and were approved by the institutional review board of Taichung Veterans General Hospital (CE13151-1). There were no statistically significant differences in age, gender, and healthcare between the sample group and all enrollees (data not shown). The database contains medical information regarding ambulatory care, inpatient care, and prescription drugs. Diagnoses were coded according to the International Classification of Disease, 9th Revision (ICD-9), which was incorporated into the database since 2000. The database used in this study can be interlinked by the scrambled unique individual's personal identification number. The NHRI safeguards the privacy and confidentiality of all beneficiaries and transfers the health insurance data to health researchers after ethical approval has been obtained. In this analysis, access to the NHIRD has been approved by the NHRI Ethics Review Committee.

### 2.2 Study population and end-point

Inclusion criteria in this study met 2 conditions: female patients with breast cancer newly diagnosed between 2001 and 2010; and age between 18 and 40. The exclusion criteria in the study group were without estrogen deprivation therapy; receiving chemotherapy or targeted therapy; receiving medications for lower urinary tract symptoms (LUTS) for more than 3 months before breast cancer diagnosis; neurologic diseases requiring 3 or more visits to outpatient clinics or 1 or more inpatient visits; and other types of cancer. The diagnosis of breast cancer was defined as code ICD-9-CM from 174.0 to 174.9. Neurologic diseases were coded ICD-9-CM from 320 to 359. Estrogen deprivation therapy, chemotherapy, target therapy, and medication for LUTS were identified by drug scientific names. The definition of LUTS was only sorted by medication used and duration of treatment for more than 1 month consecutively. Medications for OAB were trospium, imipramine, flavoxate, propiverine, oxybutynin, tolterodine, and solifenacin. In the control group, subjects who met the following criteria were also excluded: male, or uncertain gender, age below 18 or above 40; existing with cancer, coded ICD-9-CM from 140 to 208, except 195 to 199; any kinds of neurologic diseases coded ICD-9-CM from 320 to 359; receiving medication for LUTS before January 1, 2001 for more than 3 months. Incident cases of LUTS were identified from the NHIRD after the upstream patient selection.

There were 9436 subjects who met the primary inclusion criteria in the study group. After eliminating subjects who met the exclusion criteria, 1008 cases were selected as study subjects. The control group included 15,120 subjects selected randomly from a 1 million noncancer population without any neurological or psychological diseases.

In both groups, comorbidities including Charlson comorbidity index (CCI), diabetes mellitus (ICD-9-CM 250), hyperlipidemia (ICD-9-CM 272), hyperthyroidism (ICD-9-CM 410–414), chronic kidney disease (CKD; ICD-9-CM 582–583), mental diseases (ICD-9-CM 290–319, except 296 and 311), major depression (ICD-9-CM 296), and minor depression (ICD-9-CM 311) were recorded.

The use of antidepressant and antipsychotic medication for 1 month or longer were also sorted into the comparison. Urbanization and insurance amount were also included in the analysis. Urbanization may reflect the convenience of medical consultation; while insurance amount may represent the economic status of subjects.

### 2.3 Statistical analysis

The data are presented as the mean values and standard deviations for continuous variables, and proportions for categorical variables. The differences between continuous values were analyzed by using *t* test for continuous variables, and chi-squared test for categorical variables. Multivariate Cox proportional hazard regression was used to estimate the hazard ratio and 95% confidence interval for the association between the incidence of LUTS and the young female patients with breast cancer who received estrogen deprivation therapy. Propensity analysis was used for further confirming this association. The LUTS-free survival curves were plotted via the Kaplan–Meier method with statistical significance examined by the log-rank test. All statistical analyses were carried out by SAS software version 9.2 (SAS Institute, Inc., Cary, NC). A *P* value of <0.05 was considered statistically significant.

## 3 Result

**Figure 1:**
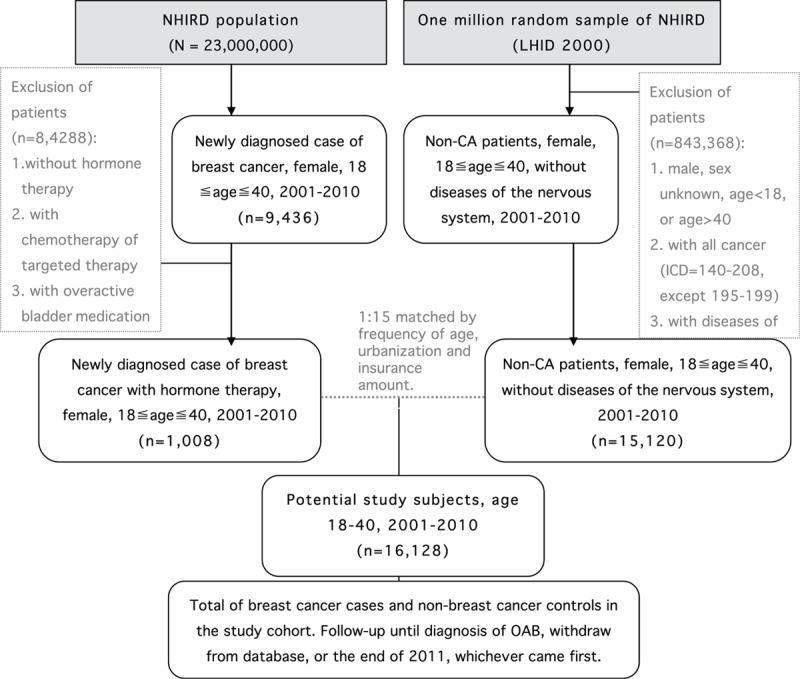
Flow diagram showing the process of overactive bladder (OAB) patient sampling and participation.

Figure 1 shows the flow diagram in patient selection and sampling. The target breast cancer group included 1008 patients. The nonbreast cancer controls in the study cohort included 15,120 cases. A comparison of these 2 groups revealed that the breast cancer study group had an older age (*P* < 0.001), higher CCI (*P* < 0.001), higher rate of mental disorder (*P* < 0.001), higher rate of major depression (*P* < 0.001), higher rate of medication with prescription drugs for major depression and psychosis (*P* < 0.001), higher rate of OAB (*P* < 0.001), and follow-up duration (*P* < 0.001). The mean age of the breast cancer cohort was 35.6 at the time of the survey. The censored OAB cases in the breast cancer group were 14 (1.4%). There were no statistically significant differences in comorbidities such as diabetes mellitus, hyperlipidemia, hyperthyroidism, CKD, urbanization, and insurance amount between 2 groups (Table 1). Table 2 shows that the subgroup analysis of OAB risk revealed a 15.93-fold increase in risk in the breast cancer group after adjustment for age and CCI. The risk of OAB increased with age and patients with breast cancer in 36 to 40 age group had a 17.46-fold greater risk of OAB than the control. Lower CCI score also had a trend of increasing risk of OAB; while CCI score 0 subgroup had a 24.87-fold increased risk of OAB. The use of antidepressants longer than 30 days had a 6.16-fold increase of OAB risk. Table 3 shows the multivariate Cox hazard analysis with adjustment with age, CCI, antidepressant or antipsychotic medications, urbanization, and insurance amounts. The breast cancer group had a 14.37-fold higher risk of developing OAB than nonbreast cancer group.

**Table 1:**
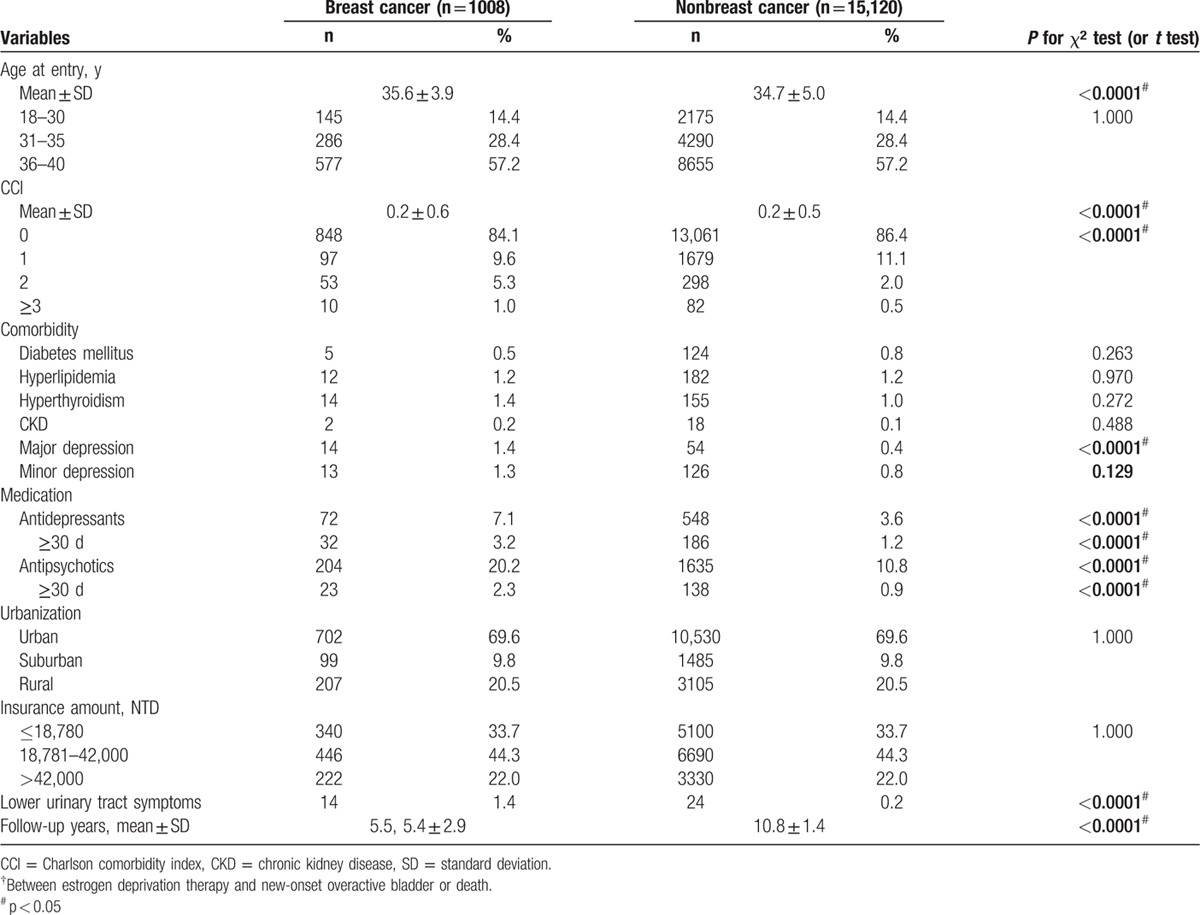
Baseline characteristics (n = 16,128).

**Table 2:**
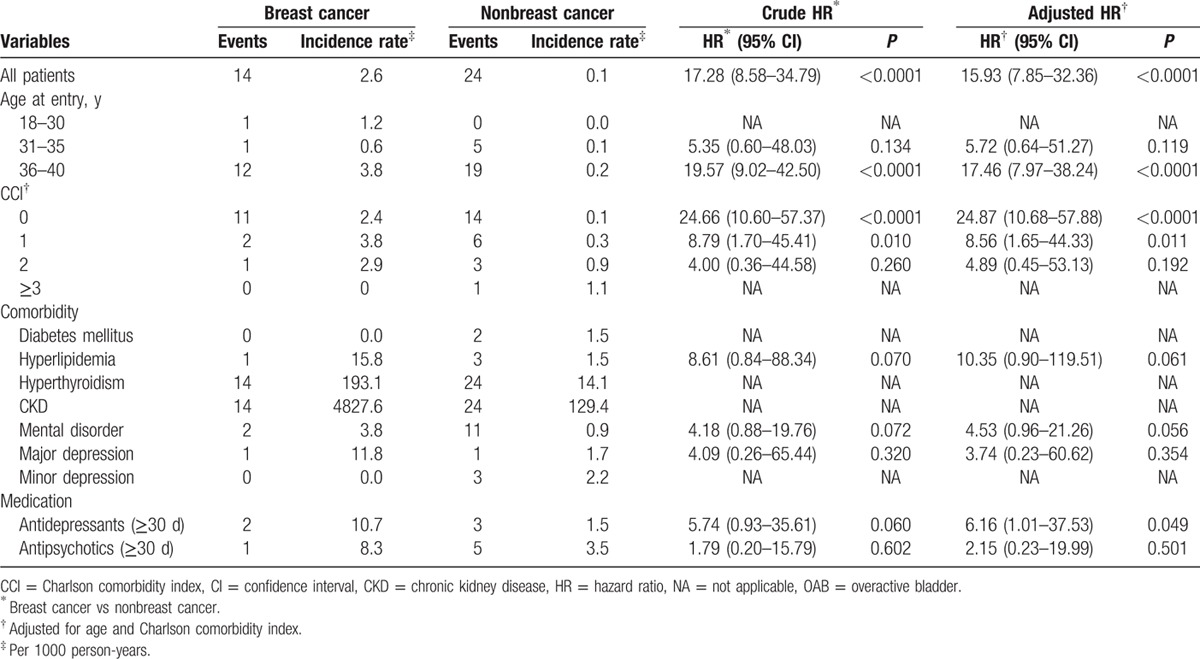
Specific subgroup analysis for new-onset OAB (n = 16,128).

**Table 3:**
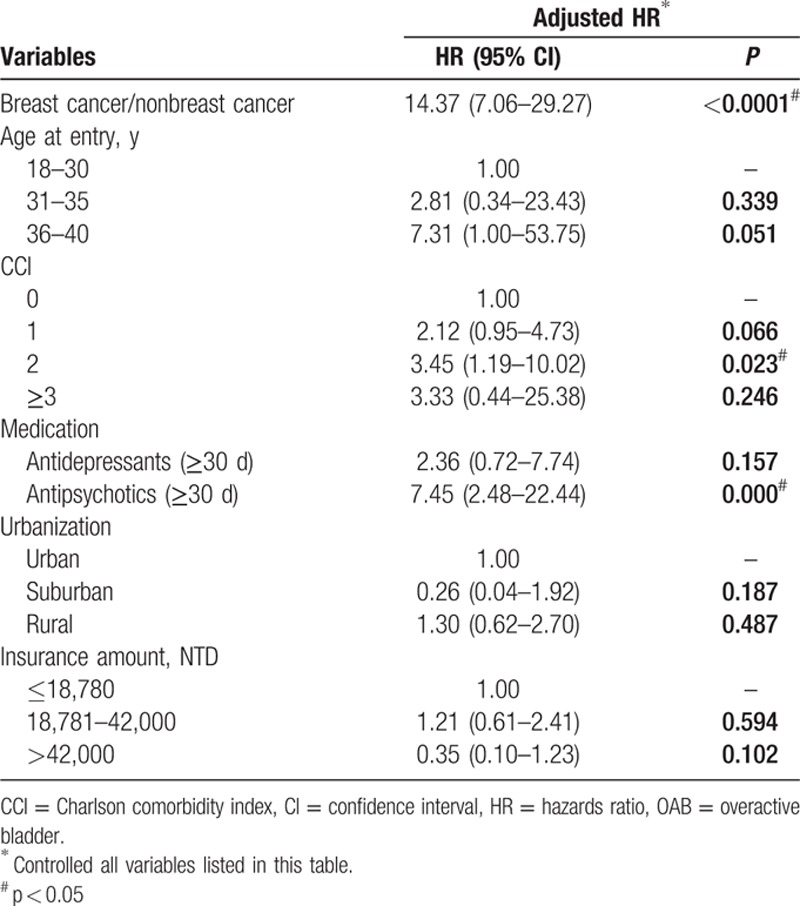
Multivariant analysis for newly onset OAB (n = 16,128).

**Figure 2:**
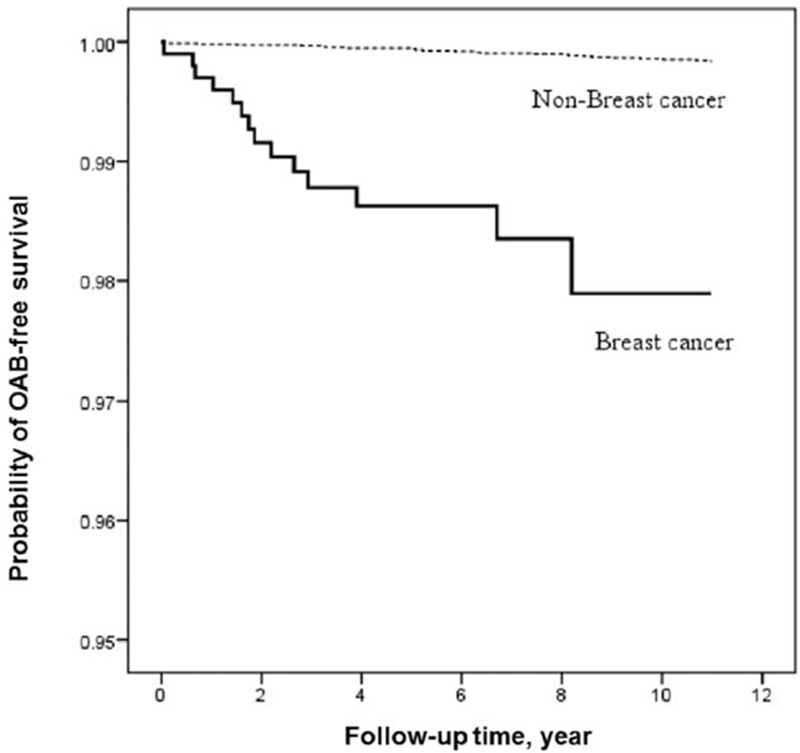
Kaplan–Meier survival analysis of probability of developing OAB with and without breast cancer (*P* < 0.001, log-rank test) (n = 16,128). OAB = overactive bladder.

Because CCI and comorbidity were highly correlated, we selected CCI for multivariate analysis. CCI score 2 had a significant 3.45-fold increase in risk of developing OAB. Similarly, mental disorders, major depression, minor depression, and medications for treatment of depression and psychosis were highly correlated. Thus, we selected antidepressant and antipsychotic drugs prescribed for longer than 30 days for multivariate analysis. Antipsychotic medication did increase the risk of OAB 7.45-fold. There was no statistically significant influence in age of entry, CCI score 1 or >3, antidepressant therapy for longer than 30 days, urbanization, or insurance amount. Figure 2 shows a time-dependent trend of the increase incidence of OAB in the breast cancer study group demonstrating abrupt increase in the first to 3 years of estrogen deprivation therapy followed by a slower increase during the next 9 years.

## 4 Discussion

Estrogen is a key steroid hormone that regulates various physiological functions in the female including the functions of the lower urinary tract.^[10,11]^

Because estrogen loss after menopause has been considered an important factor contributing to OAB symptoms,^[2,12,13]^ estrogen treatment has attracted attention as a possible method to reverse symptoms.^[13–15]^ However, over the past 2 decades there have been varying opinions about the efficacy of hormone replacement therapy in treating LUTS. Although it was believed for many years that systemic estrogen was helpful in treating LUTS, more recent epidemiological studies and meta-analyses indicate that systemic estrogen therapy increases the risk of urgency urinary incontinence (UUI)^[16–18]^ while local estrogen therapy may be beneficial for reducing UUI and OAB symptoms.^[13,14]^

Estrogen may act at multiple sites to influence lower urinary tract function because estrogen receptors have been identified in the smooth muscle of the bladder and urethra, in the urothelial lining of the bladder, in bladder nerves, and in the central nervous system.^[10,19–22]^ Animal studies showed that decreased voiding efficiency and increased postvoiding volume after OVX in rats were reversed by estrogen therapy, suggesting that hormonal changes can induce urinary frequency and impaired voiding.^[9]^ Studies in human detrusor smooth muscle revealed that estrogen in nanomolar concentrations close to the plasma levels in women induces membrane hyperpolarization which closes L-type calcium channels and suppresses muscle contractility. The hyperpolarization is due to a nongenomic mechanism that stimulates voltage and calcium-dependent potassium channels. These findings led to the proposal that altered expression and function of these ion channels may contribute to age- and sex-related changes in lower urinary tract function.^[23–26]^

Our study is the first nationwide cohort study designed to analyze the association between estrogen deprivation and female LUTS. Unlike clinical studies using precise ICD-9-CM diagnosis to define study objects, we used drug prescriptions as an indicator of our target diagnoses or disorders. According to the previously recognized low persistence rates of antimuscarinic treatment for OAB, a high drop-out rate began after 2 months of treatment.^[27]^ Thus, in our study, we defined OAB as drug therapy lasting more than 1 month. As the prescription duration increased, the comparison result was similar but the case numbers decreased.

Estrogen deprivation increased the incidence of OAB events in young females. This increase was greater in elderly than in the younger subgroup. However, the incidence of LUTS was quite low in both groups. This was no doubt due to a very strict criterion for identifying patients with LUTS. Various voiding symptoms are not uncommon in daily lives especially in women; and the etiology varies from simple urinary tract infection, postpartum pelvic organ prolapse or socioenvironmental influence. If the criterion for identifying women with OAB were less strict, for example, use of medications for <1 month, it may be confounded by some acute etiologies. If the ICD-9-CM code system is used to identify the subjects, then it has been reported that OAB is over-diagnosed by Taiwanese general physicians. The low incidence rate of 0.2% in the control group and 1.4% in the study group showed the realistic situation in Taiwanese young females (Table 1).^[2]^ Taiwanese social values promote hard work and encourage young people with health issues to maintain their daily lives rather than seeking medical help. Chen et al^[2]^ reported a 11.3% prevalence of OAB in Taiwanese young women with an age-dependent increasing pattern. Several studies in Europe and United States also showed similar results. These questionnaire-based prevalence studies also demonstrated a low treatment rate especially in young age groups.^[6,28,29]^ Our data supplemented the real world incidence in OAB treatment.

Looking into subgroup analyses, the age-dependent increasing incidence (Table 2) corresponds to other prevalence studies.^[6]^ The incidence was higher in the age range of 36 to 40, which may reflect the aging process or the influence of childbirth. In the CCI comparison, OAB occurred more often in healthy subjects than in comorbid ones and a higher CCI did not increase the risk. There were only 2 patients in control, no breast cancer group found to have diabetes mellitus. This was a very low rate compared with a previous prevalence study.^[30]^ This suggests that urological care of patients who have diabetes mellitus in Taiwan should be more aggressive. Interestingly, all subjects with OAB in both groups had a diagnosis of hyperthyroidism or CKD which implies the potential association of female OAB with some metabolic diseases.^[31]^ This finding is also consistent with previous studies of Ho et al^[31]^ in Taiwanese populations. Depression and psychosocial distress may co-exist in both cancer and OAB patients.^[32]^ It has been reported that OAB may induce depression and anxiety with a symptom-severity-dependent pattern.^[5]^ Postpartum depression has been reported to increase risk of female OAB.^[33]^ However, after adjustment for antidepressant therapy, we can still observe a higher risk of OAB in female patients with breast cancer who received estrogen deprivation therapy.

Using multivariate analysis to adjust for all possible variables the breast cancer group still had a 14.37-fold risk to develop OAB (Table 3). CCI score still had an impact to increase risk of OAB and this may correspond to epidemiologic studies about the association of chronic diseases and female OAB.

The Taiwanese health insurance system covers medical care for most health-related problems and is distributed all over the island. Therefore, it is not difficult for an individual to obtain a consultation or treatment. This is consistent with the multivariant analysis, which showed no statistical differences in urbanization and insurance amounts.

The proportion of OAB-free survival also showed a time-dependent pattern that indicated a longer estrogen deprivation is linked with a higher incidence of OAB. Although few subjects exhibited OAB early in the estrogen deprivation therapy, the steady increase in the beginning 3 years showed the possible patient-oriented variation rather than a confounding bias. In addition, the rapid increase in OAB development flattened after 3 years. This might influence the treatment of clinical postmenopause conditions suggesting the importance of estrogen supplement to treat female OAB only in the beginning years. The estrogen deprivation time-dependent pattern (Fig. 2) also corresponds to prevalence studies in aging females.^[6]^

There were still some limitations and confounding factors in our study. Patients with breast cancer have the potential for greater numbers of medical consultations and hence an increased likelihood of diagnosis and treatment of OAB. The prescribed hormone deprivation therapy may also be a factor in inducing OAB, although similar adverse events were not seen in most clinical trials.^[34,35]^ The marriage status which may influence the psychosocial condition of women could not be obtained in the database. We can only adjust for this based on the diagnosis of psychological, mental health, or antidepressant prescriptions. Also, the partum status of subjects was lacking. Postpartum stress or pelvic structure changes have a direct impact on female voiding even though the influence is often temporary. The definition of female OAB defined by prescribed drugs and duration of treatment made the overall case numbers small. Furthermore the age limit was planned to eliminate the menopause interference though it largely decreased the case numbers. From this method, we can only identify OAB subjects based on medical treatment rather than the information about core symptoms or the real clinical diagnoses.

In conclusion, we identified a strong association of estrogen deprivation and incidence of OAB in young females. This study emphasizes the importance of estrogen in maintenance of female normal voiding function clinically and is consistent with previous epidemiological and animal studies.
